# Association of Physical Activity Patterns with the Metabolic Syndrome in Korean Adults: A Nationwide Cross-Sectional Study

**DOI:** 10.31083/j.rcm2504115

**Published:** 2024-03-26

**Authors:** Seung Woo Shin, Junghoon Kim

**Affiliations:** ^1^Sports and Exercise Medicine Laboratory, Korea Maritime & Ocean University, 49112 Busan, Republic of Korea; ^2^School of Applied Health, Cal Poly Humboldt, Arcata, CA 95521, USA

**Keywords:** weekend warriors, moderate to vigorous physical activity (MVPA), metabolic syndrome (MetS), physical inactivity

## Abstract

**Background:**

Recent research has focused on a new group called the “weekend warriors”. These individuals accumulate their recommended moderate to vigorous physical activity (MVPA) over just 1–2 days, often during weekends, while remaining relatively inactive during the rest of the week. However, the effects of engaging in low-frequency MVPA on the risk of metabolic syndrome (MetS) are not well understood. This study investigated the association between physical activity patterns and the risk of MetS among Korean adults.

**Methods:**

This study included 26,197 participants (11,804 male and 14,393 female) aged ≥20 years from the Korea National Health and Nutrition Examination Survey. MVPA was measured using a global physical activity questionnaire. MetS was defined as the presence of more than three risk factors.

**Results:**

The odds ratio (OR) for MetS was 0.60 (95% confidence interval [CI] = 0.52, 0.70) in the “regularly active” group and 0.82 (95% CI = 0.69, 0.98) in the “weekend warrior” group compared to that in the inactive group (reference), which controlled for all covariates. For sensitivity analyses, the results across all subgroups exhibited similar patterns, with more pronounced effects observed in women, middle-aged individuals, and non-drinkers/light drinkers.

**Conclusions:**

Our findings suggest that concentrated bouts of moderate to vigorous physical activity, even if undertaken infrequently, confer health benefits that align with the recommended guidelines. This study contributes to the growing evidence on the relationship between physical activity patterns and MetS risk in Korean adults. The study also emphasizes the potential of different activity patterns in mitigating metabolic risk.

## 1. Introduction

Physical inactivity is a significant contributor to the global burden of non-communicable diseases [1], particularly metabolic syndrome (MetS), which is a complex constellation of cardiometabolic risk factors associated with an increased risk of cardiovascular diseases and type 2 diabetes [1]. The prevalence of MetS is increasing in South Korea and poses a critical public health challenge. According to recent data from the Korean National Health and Nutrition Examination Survey (KNHANES), the prevalence of MetS among Korean adults reached 22.9% (male: 27.9%, female: 17.9%) in 2018, underscoring the urgency for effective interventions [2].

Despite the well-established benefits of regular physical activity, adherence to the recommended guidelines remains a challenge [3]. The international guidelines recommend engaging in at least 150 min of moderate to vigorous physical activity (MVPA) per week to maintain optimal health and prevent chronic diseases [4]. However, many individuals struggle to meet these guidelines because of various barriers, such as time constraints and work commitment [3, 5]. Recent research has focused on an emerging group called the “weekend warriors”. These individuals accumulate their recommended MVPA over just 1–2 days, often during weekends, while remaining relatively inactive during the rest of the week [6, 7, 8]. However, the effects of engaging in low-frequency MVPA on the risk of MetS are not well understood.

Several seminal studies have investigated the health implications of the “weekend warrior” phenomenon. Khurshid *et al*. (2023) [7] demonstrated that “weekend warriors”, using accelerometer-derived physical activity data, had a lower incidence of cardiovascular disease than their inactive counterparts. Similarly, another meta-analysis conducted by Dos Santos *et al*. (2022) [6] found that individuals who engaged in MVPA only on weekends exhibited lower risks of all-cause and cause-specific mortality than those who remained completely inactive. These findings collectively suggest that concentrated bouts of MVPA, even if undertaken infrequently, confer health benefits that align with the recommended guidelines [9]. Despite the increasing prominence of weekend warrior behavior and its potential implications for metabolic health, there is a lack of research specifically examining this pattern in the Korean population. While international studies have explored the health benefits of weekend warrior behavior, there is a critical need to investigate its prevalence and impact within the unique culture and lifestyle of South Korea.

Therefore, this study aimed to investigate the associations between physical activity patterns and risk of MetS among Korean adults using data from KNHANES 2017–2019.

## 2. Materials and Methods

### 2.1 Participants

**Fig. 1.**
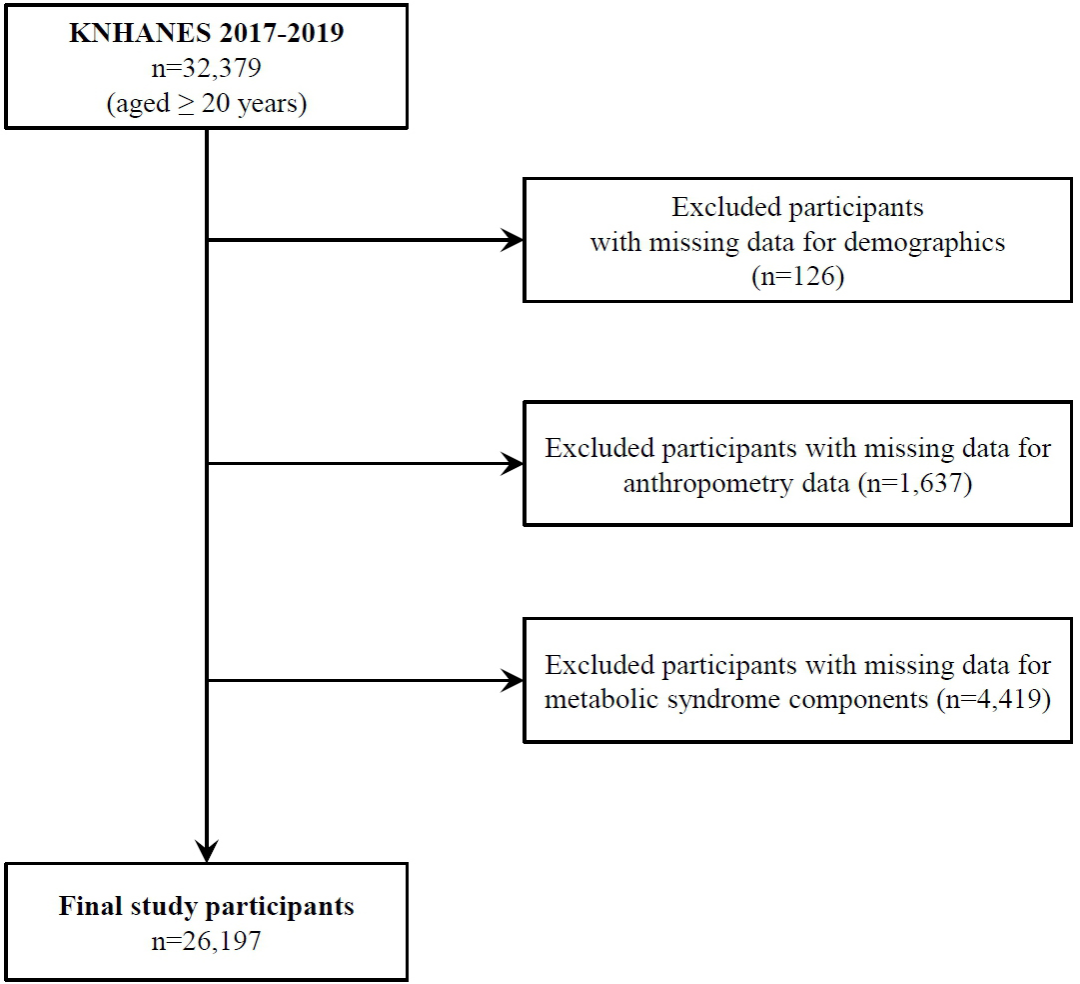
**Study participants flow diagram**. KNHANES, Korean National Health and Nutrition Examination Survey.

This study was conducted on adults aged 20 years and older from the KNHANES dataset conducted between 2017 and 2019 [10, 11]. Of the 32,379 participants, we excluded 126 with missing demographics and 1637 with missing anthropometric data. Finally, we also excluded 4419 participants who did not fast for at least 8 h at the time of blood sampling for the diagnosis of MetS. After excluding participants with missing data, 26,197 were included in the final analysis (Fig. 1). The KNHANES received research ethics approval from the Korean Agency for Health and Welfare Affairs, and all participants provided written informed consent.

### 2.2 Physical Activity

Physical activity was measured using the Global Physical Activity Questionnaire (see **Supplementary Materials**), which examined the time spent in MVPA during leisure time and work-related physical activity [12]. To analyze the effect of patterns on the frequency of leisure-time physical activity participation, this study examined the number of days that participants engaged in at least 10 min of MVPA and the duration of each activity in minutes.

### 2.3 Physical Activity Pattern Classifications

For this study, we categorized participants as “active” if they met the World Health Organization (WHO) physical activity guidelines of 150 min or more of moderate-intensity activity per week, 75 min or more of vigorous-intensity activity per week, or 150 min or more of MVPA per week combined [4]. In addition, to categorize participants according to the frequency of physical activity participation, those who participated in MVPA only 1–2 days per week were categorized as “weekend warriors” and those who participated 3–7 days per week as “regular active” [6]. Participants who did not meet the physical activity guidelines were categorized as “inactive”.

### 2.4 MetS/Cardiometabolic Syndrome

The abdominal circumference was measured three times in cm, and the average value was used. Blood pressure was measured three times in a sitting position after at least 5 min of rest, and the minimum value was used for the analysis. Blood samples were collected after fasting for at least 8 h, and fasting blood glucose, triglyceride (TG), and high-density lipoprotein cholesterol (HDL-C) levels were analyzed using the enzyme method.

We considered waist circumference (cm), systolic and diastolic blood pressure (mmHg), fasting blood glucose (mg/dL), TG (mg/dL), and high-density lipoprotein cholesterol (mg/dL) as risk factors for MetS [2, 13]. Abdominal obesity was defined as a waist circumference ≥90 cm for men and ≥80 cm for women. Hypertension was defined as systolic blood pressure ≥130 mmHg and/or diastolic blood pressure ≥85 mmHg or taking medication for hypertension. Hyperglycemia was defined as a fasting (>8 h) blood glucose level ≥100 mg/dL or taking medication for hyperlipidemia. Low HDL-C levels were defined as <40 mg/dL in men and <50 mg/dL in women. Finally, we calculated the components of MetS (0–5) and defined MetS as having three or more risk factors [2].

### 2.5 Confounding Factors

Several variables were used as confounders, including sociodemographic factors such as age, sex, education level, household income, smoking status, alcohol consumption, and energy intake. Education level was categorized into three groups: <high school, high school, and >high school; household income was categorized into four groups using quartiles; smoking status was categorized as never, past, and current; and alcohol consumption was categorized as never, once a week, two to four times a week, and four or more times a week. The body mass index was calculated as weight/height (${\mathrm{kg/m}}^{2}$). Total energy intake (kcal/day) was calculated using calorie intake data from a 24-h dietary recall survey.

### 2.6 Statistical Analysis

All data analyses were conducted using stratified, random, and cluster sampling of a complex survey design. All statistical analyses were performed using the R software package (version 3.0.4, R Core Team, Vienna, Austria) [14], and the statistical significance level was set at *p* < 0.05. Continuous variables, such as age and physical activity time, were presented as weighted means and standard errors, and categorical variables, such as MetS components and MetS, were presented as weighted percentages. To compare the amount of MVPA according to physical activity patterns, a survey linear regression analysis was conducted, and the prevalence of MetS and its components were analyzed using the chi-square test.

The risk of MetS according to physical activity patterns was analyzed using a survey logistic regression. Odds ratios (OR) and 95% confidence intervals (CI) for MetS were calculated for the “weekend warrior” and “regular active” groups and compared with the “inactive group” (reference).

We also created three models to consider the effects of the confounding variables. In Model 1, we analyzed the association between physical activity patterns and the risk of MetS without covariates, whereas in Model 2, we adjusted for age and sex. In Model 3, we adjusted for age, sex, education, household income, smoking status, alcohol consumption, and energy intake.

In the sensitivity analysis, we performed a logistic regression analysis by age group, sex, smoking status, and alcohol consumption. All results are presented based on Model 3.

## 3. Results

The characteristics of the participants based on their physical activity patterns are presented in Table 1. The mean age of the participants was 44.1 ± 0.2 years, with the regularly active group having a significantly lower mean age. There were significant differences in sex, education, household income, drinking habits, and smoking status based on physical activity patterns (all *p* < 0.001).

**Table 1.** **Participants characteristics by physical activity patterns**.

		Physical activity ${\mathrm{pattern}}^{\text{a}}$				*p*-value
		Overall (N = 26,197)	Inactive (N = 22,647)	Regularly active (N = 2186)	Weekend warrior (N = 1364)	
Age ${\mathrm{(years)}}^{\text{b}}$		44.1 ± 0.2	44.7 ± 0.2	39.4 ± 0.4	43.7 ± 0.4	<0.001
Body mass index (${\mathrm{kg/m}}^{2}$)		23.7 ± 0.03	23.6 ± 0.04	24.0 ± 0.10	24.1 ± 0.10	<0.001
Energy intake (kcal/day)		2009.5 ± 9.5	1981.9 ± 9.9	2181.9 ± 27.6	2217.7 ± 37.5	<0.001
${\mathrm{Sex}}^{\text{c}}$						<0.001
	Male	11,804 (50.4)	9741 (47.8)	1244 (63.0)	819 (66.3)	
	Female	14,393 (49.6)	12,906 (52.2)	942 (37.0)	545 (33.7)	
Education level						<0.001
	<High school	9014 (27.9)	8502 (31.0)	326 (11.7)	186 (9.6)	
	High school	7488 (31.2)	6243 (30.2)	773 (37.2)	472 (34.8)	
	>High school	9695 (40.9)	7902 (38.8)	1087 (51.1)	706 (55.6)	
Household income						<0.001
	Q1	4774 (15.0)	4463 (16.3)	222 (9.7)	89 (4.8)	
	Q2	6440 (24.1)	5775 (25.2)	422 (18.4)	243 (17.7)	
	Q3	7190 (29.2)	6170 (29.1)	603 (28.0)	417 (32.1)	
	Q4	7793 (31.7)	6239 (29.4)	939 (43.9)	615 (45.4)	
Alcohol consumption						<0.001
	Never	8638 (28.8)	8005 (31.2)	393 (16.1)	240 (15.0)	
	Once a week	12,312 (49.8)	10,251 (47.9)	1291 (61.8)	770 (57.6)	
	2–3 times/week	3615 (15.2)	2939 (14.4)	389 (17.3)	287 (22.8)	
	≥4 times/week	1632 (6.2)	1452 (6.5)	113 (4.8)	67 (4.6)	
Smoking status						<0.001
	Never	17,199 (63.0)	15,122 (64.2)	1324 (58.9)	753 (51.6)	
	Former	4717 (17.8)	3848 (16.6)	508 (22.3)	361 (27.1)	
	Current	4281 (19.2)	3677 (19.2)	354 (18.8)	250 (21.3)	

^a^The physical activity patterns were classified as follows: inactive (MVPA <150 min/wk), weekend warrior (≤2 sessions/wk), or regularly active (≥3 sessions/wk). ^b^Weighted mean ± standard error (all such values). ^c^Frequency and weighted percentages (all such values). *p-*values were calculated using *t*-test for continuous variables and chi-square test for categorial variables. Abbreviation: MVPA, moderate to vigorous physical activity.

**Fig. 2.**
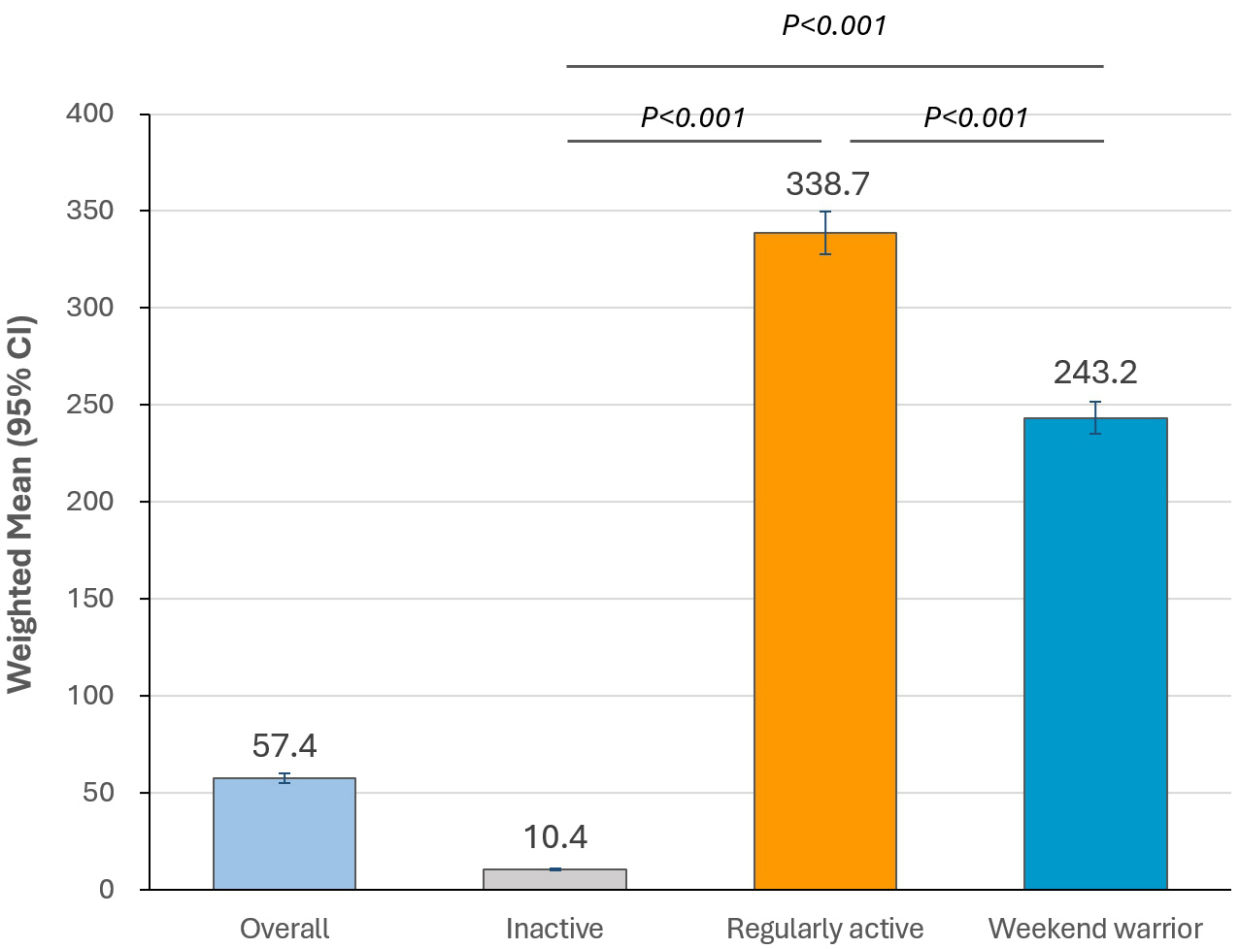
**Time spent in moderate to vigorous physical activity according to physical activity patterns**. *p*-values were calculated using the survey regression model. All values are presented as weighted means with 95% confidence intervals.

The time spent on MVPA according to physical activity patterns is illustrated in Fig. 2. Time spent on moderate to vigorous activities was significantly higher in the “regularly active” group than in the “inactive” and “weekend warrior” groups (all *p* < 0.001).

**Fig. 3.**
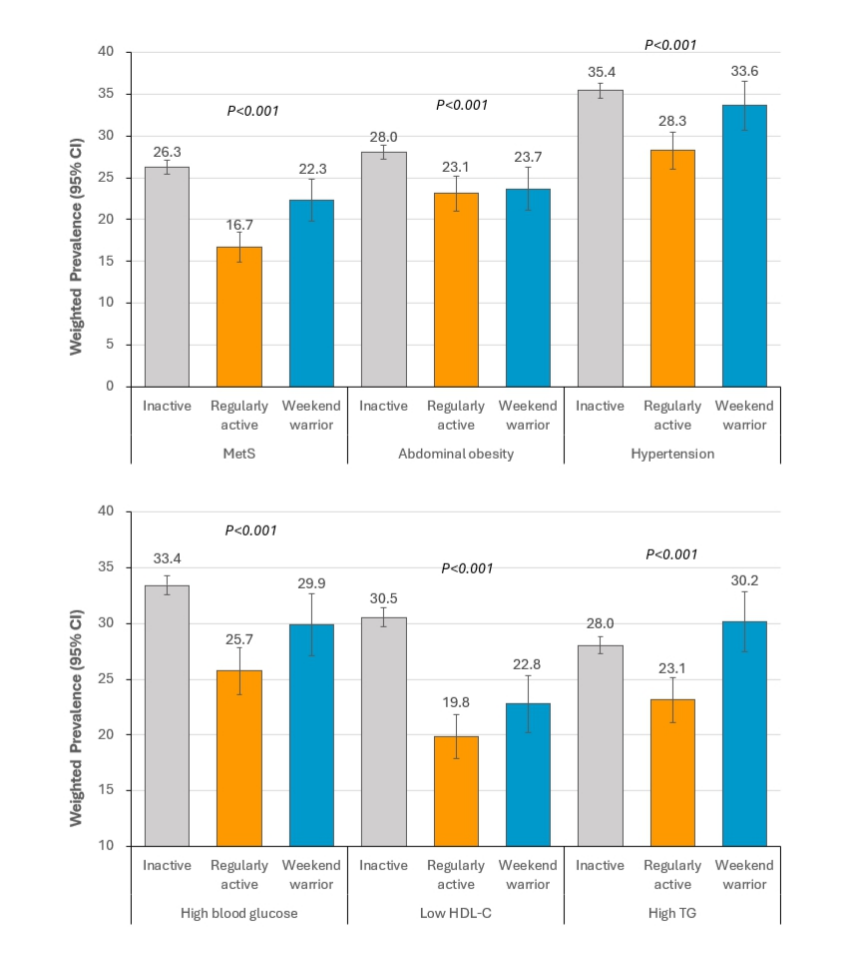
**Prevalence of metabolic syndrome and its components according to physical activity patterns**. *p*-values were calculated using the Rao–Scott chi-square test for weighted samples. All values were presented as weighted percentage ±95% confidence intervals. MetS, metabolic syndrome; TG, triglyceride; HDL-C, high-density lipoprotein cholesterol.

The prevalence of MetS and its components based on physical activity patterns are displayed in Fig. 3. The prevalence of MetS was significantly higher in the “inactive” group (26.3%) than in the “regularly active” group (16.7%) and the “weekend warrior” group (22.3%). Similar patterns were observed for abdominal obesity, hyperglycemia, and low HDL-C levels but not for hypertension and high TG levels (Fig. 3).

The ORs and 95% CIs for the association between physical activity patterns and MetS risk are presented in Table 2. In Model 1, the OR for MetS was 0.56 (95% CI = 0.49, 0.64) in the “regularly active” group and 0.80 (95% CI = 0.69, 0.94) in the “weekend warrior” group compared to that in the “inactive” group (reference). These results remained consistent in Model 2, which was adjusted for sex and age, and in Model 3, which controlled for all covariates (Table 2).

**Table 2.** **Associations of physical activity patterns and the risk of metabolic syndrome (n = 26,197)**.

	Physical activity ${\mathrm{pattern}}^{\text{a}}$, adjusted OR (95% CI)					
	Inactive		Regularly active		Weekend warrior	
Model $1^{\text{b}}$	1.00	[Reference]	0.56	(0.49, 0.64)***	0.80	(0.69, 0.94)**
Model $2^{\text{c}}$	1.00	[Reference]	0.65	(0.57, 0.75)***	0.80	(0.68, 0.94)**
Model $3^{\text{d}}$	1.00	[Reference]	0.60	(0.52, 0.70)***	0.82	(0.69, 0.98)*

^a^The physical activity patterns were classified as follows: inactive (MVPA <150 min/wk), weekend warrior (MVPA ≥150 min/wk with ≤2 sessions/wk), or regularly active (MVPA ≥150 min/wk with ≥3 sessions/wk). ^b^Unadjusted. ^c^Adjusted for age and sex. ^d^Model 2 + education level, household income, smoking status, alcohol consumption, energy intake, and body mass index. *p-value < 0.05; **p-value < 0.01; ***p-value < 0.001. Abbreviations: OR, odds ratio; CI, confidence interval; MVPA, moderate to vigorous physical activity.

**Fig. 4.**
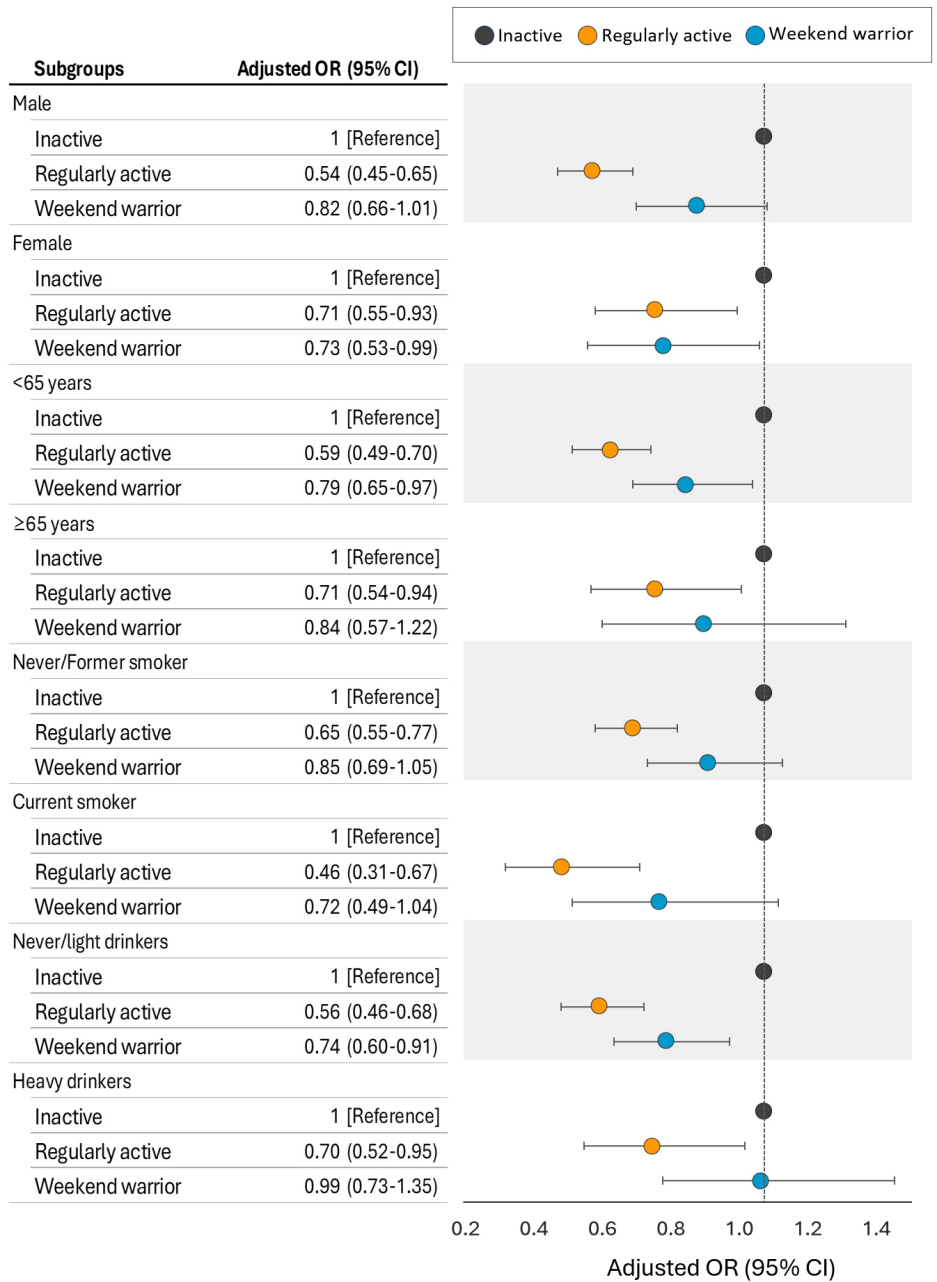
**Adjusted odds ratios (ORs) (95% confidence interval (CI)) for metabolic syndrome according to physical activity patterns by subgroups**. Adjusted for age (continuous), sex, education level, household income, smoking status, alcohol consumption, energy intake, and body mass index, subgroup variables were not included in the models as covariates in each analysis.

For sensitivity analyses, subgroup analyses were conducted based on sex, age, and smoking and drinking habits (Fig. 4). These subgroup analyses were performed using Model 3, which accounted for all covariates. The results across all subgroups exhibited a similar pattern, with more pronounced effects observed in women, middle-aged individuals, and non-drinkers/light drinkers.

## 4. Discussion

This study investigated the association between different physical activity patterns and the prevalence of MetS in a Korean population. Individuals classified as “regularly active” and “weekend warriors” exhibited a reduced risk of MetS, and these associations remained robust even after adjusting for potential confounding factors. Our findings revealed distinct patterns in the ORs for MetS among the different subgroups, shedding light on the potential protective effects of specific physical activity behaviors.

The “regularly active” group emerged as a prominent contributor to a reduced MetS risk across various demographic categories. This group consistently demonstrated lower ORs for MetS, regardless of sex, age, or subgroup classification. These results are consistent with previous studies highlighting the positive impact of regular physical activity on metabolic health [15, 16, 17, 18]. The persistent association between regular physical activity and reduced risk of MetS underscores the significance of maintaining a consistent physical activity routine and transcending demographic boundaries. This evidence builds on previous findings demonstrating that regularly active individuals have the lowest risk of developing MetS.

The “weekend warrior” group exhibited a significant association with lower ORs for MetS across the entire cohort. This finding suggests that individuals who accumulate their recommended physical activity during concentrated time periods such as weekends experience a favorable impact on their metabolic health. However, the benefits of low-frequency physical activity are not well understood. One of the most striking findings of this study was that participating in one to two sessions of moderate-intensity physical activity per week may be sufficient to reduce the risk of MetS. In a study involving 13,505 women and 6997 men, Xiao *et al*. [19] found that compared to that for inactive participants, the OR for diabetes was 0.48 (95% CI, 0.32–0.73) for weekend warriors, 0.37 (95% CI, 0.0.29–0.48) for regularly active participants, and 0.65 (95% CI, 0.40–1.04) for those who were insufficiently active.

This study also expands previous research on the association between weekend warrior physical activity patterns and mortality due to cardiovascular disease and cancer [6]. However, while these findings suggest a potential benefit of the “weekend warrior” lifestyle, further research is needed to fully understand the implications of these physical activity patterns on long-term health outcomes.

Voluntarily chosen physical activity during leisure time, including walking, running, and participating in sports, tends to be purposeful and of moderate to vigorous intensity. This study found that “regularly active” participants and “weekend warriors” had a lower risk of MetS than “inactive” participants, suggesting that the frequency and duration of physical activity are not as critical for those who meet the physical activity guidelines. Nevertheless, in this study, regular physical activity was associated with the lowest risk of MetS prevalence; regular physical activity reduced the MetS risk by approximately 40% compared to that in the “inactive” group but only by approximately 18% in the “weekend warrior” group. The “regularly active” group achieved the recommended physical activity frequency of three or more times per week, resulting in a higher total MVPA than the “weekend warriors”. In this study, the “regularly active” group engaged in approximately 54 min of moderate-intensity and 42 min of vigorous-intensity activity compared with the “weekend warrior” group. Many previous studies have reported a dose-response relationship between physical activity, risk of metabolic diseases, and mortality [20]. These differences in total physical activity could explain the observed variations in MetS risk [19].

In the present study, the subgroup analyses of the association between weekend warrior physical activity patterns and MetS prevalence showed overall consistent patterns. However, statistically significant associations were found in female, middle-aged (<65 years), and non-drinking/light drinking groups. The significant association between weekend warrior physical activity patterns and reduced risk of MetS in women and middle-aged individuals highlights the potential relevance of these patterns in specific populations. These findings underscore the importance of tailoring physical activity recommendations to different demographic profiles while considering individual constraints and preferences. Moreover, achieving the recommended amount of physical activity with 1–2 sessions per week likely requires either prolonged bouts of activity or relatively high-intensity exercise per session. Therefore, this activity pattern may be more suitable for younger individuals than older individuals.

Physical inactivity is responsible for approximately 25% of premature deaths worldwide and incurs substantial healthcare costs of at least $54 billion annually [21]. Physical activity recommendations encompass a range of factors, including frequency, duration, and intensity. Regular physical activity is advised to manage body weight, cholesterol levels, and blood pressure.

This study demonstrated that less frequent physical activity, which is more manageable for individuals with busy lifestyles, offers significant metabolic health benefits. In our country, millions of people engage in physical activities such as running, biking, or biking at least once a week. Although “weekend warriors” engage in vigorous-intensity physical activities, the quality of these activities may be more critical than their quantity. For example, running, a popular form of vigorous-intensity physical activity, reduces the risk of MetS, even in low amounts [22]. High-intensity exercise enhances cardiorespiratory fitness more effectively than an equivalent amount of moderate-intensity exercise [23]. Cardiorespiratory fitness is a stronger predictor of MetS than physical activity alone [24, 25]. In a classic series of experiments, a recent systematic review demonstrated that cardiorespiratory fitness could be maintained with just two bouts of high-intensity exercise per week [26].

However, high-intensity physical activity, when performed simultaneously, may increase the risk of physical activity-related musculoskeletal injuries [27], particularly in sedentary individuals. This study suggests that meeting physical activity guidelines, even at a low frequency of once or twice a week, helps prevent metabolic diseases. However, further research is required to understand the potential risk of injury. Most international physical activity guidelines do not specify a recommended frequency [4]; however, they advise inactive adults to gradually increase both duration and frequency before intensity to achieve recommended activity levels while reducing the risk of injuries [28, 29].

### Strengths and Limitations

A notable strength of our study lies in the use of data from the KNHANES, a representative dataset encompassing a large and diverse sample of the Korean population. Using this extensive dataset allowed us to capture a comprehensive snapshot of physical activity patterns and their potential effects on the prevalence of MetS across various demographic groups. This enhances the generalizability of our findings, making them applicable to the broader Korean population.

However, our study has some limitations. This cross-sectional design prevented us from establishing causality between physical activity patterns and the risk of MetS. Longitudinal studies are warranted to delineate the temporal relationships and better comprehend the direction of influence. Additionally, the reliance on self-reported physical activity data introduces the possibility of recall bias and misclassification into activity groups. The inclusion of objective measurements, such as accelerometry, would strengthen the accuracy of our findings. Although we did not evaluate the influence of occupational physical activity in this study, we considered it a potential covariate because it differs from leisure-time physical activity in that it is discretionary. In this study, we defined “weekend warriors” as individuals meeting physical activity guidelines with a frequency of 1–2 times per week. However, we lacked precise information regarding the specific days the participants engaged in physical activity. Additionally, while we assessed patterns of physical activity frequency, we could not determine whether these activities occurred on consecutive or non-consecutive days. Further research is needed to investigate whether these engagement patterns, independent of total physical activity, impact MetS. Furthermore, potential confounding variables such as genetic factors were not comprehensively addressed in our analysis. We also controlled for demographic factors and various covariates known to influence MetS, including smoking, drinking habits, and dietary intake. However, the categorization of drinking habits based solely on frequency within the past month might not fully capture potential effects, as it does not account for the amount of alcohol consumed.

## 5. Conclusions

This study contributes to the growing evidence on the relationship between physical activity patterns and MetS risk in Korean adults. We found that the lower risk for MetS in both the “regularly active” and “weekend warrior” groups emphasizes the potential for different activity patterns to mitigate metabolic risk. This robust study of the general Korean population provides evidence of a significantly lower risk of MetS among “weekend warriors” than among inactive individuals.

## Data Availability

The data sets generated and/or analyzed during the current study are available in the Korea National Health and Nutrition Examination Survey repository, https://knhanes.kdca.go.kr/knhanes/.

## Abbreviations

CI, confidence interval; HDL-C, high-density lipoprotein cholesterol; KNHANES, Korean National Health and Nutrition Examination Survey; MVPA, moderate to vigorous physical activity; MetS, metabolic syndrome; OR, odds ratio; TG, triglyceride.

## Supplementary Material

Supplementary material associated with this article can be found, in the online version, at https://doi.org/10.31083/j.rcm2504115.
