# Supplementary material for: Association of Physical Activity Patterns with the Metabolic Syndrome in Korean Adults: A Nationwide Cross-Sectional Study
Source: Rev Cardiovasc Med. 2024 Mar 26;25(4):115. doi: 10.31083/j.rcm2504115 (PMC11262443; doi:10.31083/j.rcm2504115)
Supplement: Supplementary file 1 [file 2153-8174-25-4-115-s1.docx]

**Supplementary Materials**

The Global Physical Activity Questionnaire (GPAQ), used in the Korean National Health and Nutrition Examination Survey, was developed by the World Health Organization (WHO) and is available for download on the WHO website.

**Global physical activity questionnaire (GPAQ)**

<https://www.who.int/publications/m/item/global-physical-activity-questionnaire>
